# Improving Patient-Centered Screening for Diabetes and Follow-Up in a Rural Primary Care Clinic

**DOI:** 10.1016/j.aed.2026.04.019

**Published:** 2026-06-21

**Authors:** Michelle M. Anderson-Boyd, Kimberly A. Couch

**Affiliations:** 1 Strawberry Wilderness Community Clinic, John Day, Oregon; 2 Frontier Nursing University, Versailles, Kentucky

**Keywords:** diabetes, guidelines, patient-centered, quality improvement, rural

## Abstract

**Objective:**

Diabetes is a significant health problem that has reached pandemic proportions and affects 830 million people worldwide. In 2022, the direct and indirect costs associated with diabetes in the United States were $412.9 billion. On average, people with diagnosed diabetes have medical expenditures 2.6 times higher than would be expected in those without diabetes. A chart audit at a rural primary care clinic revealed that only 44% of the population with diabetes had proper care at the time of diagnosis. This quality initiative aimed to improve patient-centered diabetes screening and follow-up in an 8-week period to 80%.

**Case Report:**

A rapid Plan-Do-Study-Act process approach was implemented for the initiative in four 2-week cycles over 8 weeks. Results from each cycle dictated the test of change for the subsequent cycle. A diabetes screening tool and a patient engagement toolkit were adapted from updated, evidence-based guidelines to influence adequate and appropriate screening while actively engaging a population of patients with diabetes.

**Discussion:**

Over the 8-week period, 128 charts were screened, and the rate of patient-centered diabetes follow-up improved from 44% to 83%. In that same period, 201 patients were screened, and the rate of diabetes screening improved by 50%.

**Conclusions:**

This initiative improved patient-centered diabetes screening and follow-up by implementing evidence-based screening tools and adapted engagement toolkits that originated from the American Diabetes Association. Further research should explore how early intervention and effective management of prediabetes and diabetes impact the development of comorbidities associated with the disease.

**Highlights**

• Diabetes is a major global health concern affecting 830 million people worldwide
• Chart audits in a rural primary care clinic found only 44% of patients received appropriate care at the time of diagnosis of prediabetes or diabetes
• A rapid Plan-Do-Study-Act approach was implemented over four 2-week cycles to improve screening and follow-up
• Evidence-based diabetes screening and patient engagement tools improved patient-centered care, increased team satisfaction, demonstrated potential sustainability, and showed adaptability for implementation across diverse health care settings

**Clinical Relevance**

Improving diabetes screening and structured follow-up in rural primary care supports earlier diagnosis, timely intervention, and adherence to evidence-based management. These improvements can reduce progression from prediabetes to diabetes, improve glycemic outcomes, decrease complications, and strengthen long-term population health outcomes.

Diabetes is a chronic health condition that has had a substantial global health impact and is currently the eighth leading cause of death in the world (World Health Organization.^1^ In the United States, the total annual cost of diabetes in 2022 was $412.9 billion, including $306.6 billion in direct medical costs and $106.3 billion in indirect costs.^2^ In Grant County, Oregon, 9% of the adult population has diabetes, which is consistent with the national average.^3^

A review of data collected via chart audits, patient surveys, and direct patient tracer analysis at Strawberry Wilderness Community Clinic (SWCC) in Grant County, Oregon, revealed that only 44% (*n* = 12 of 27) of patients newly diagnosed with prediabetes or diabetes (hemoglobin A1c [HbA1c] ≥5.7%) were advised on a follow-up plan of care. Further analysis revealed that only 23% (*n* = 13 of 56) of patients were properly screened for diabetes with a standardized screening tool. A preinitiative survey of patients revealed a satisfaction baseline of 2.5 on a Likert scale (1 = *very dissatisfied* to 5 = *very satisfied*), indicating overall dissatisfaction in care regarding disease management. When surveyed preinitiative, 27% (*n* = 4 of 15) of the clinical staff did not understand their role in patient education regarding diabetes.

## Available Knowledge

Per the American Diabetes Association^4^ clinical guidelines, all individuals should be screened for diabetes risk factors and tested with standardized testing if they meet the criteria. Neu et al^5^ recommend that organizations establish a diabetes registry to track screening compliance at appropriate screening intervals and follow-up based on patient history and comorbidities. Kidanemariam et al^6^ concluded that it is vital to have clinician-patient collaboration when developing care plans to ensure plan implementation, effectiveness, and compliance.

### Rationale

The patient-centered domain represents the provision of respectful and considerate care based on individual needs and preferences. Diabetes is a challenging disease to manage and can result in comorbidities without patient abidance. According to Nurchis et al^7^, patient-centered care not only improves patient outcomes but also has the potential to ease health care processes and reduce health costs in clinical settings. Shared decision-making during clinical visits was also used to support interventions through evidence based screening and patient engagement. In their study, ElSayad et al^8^ showed that to engage patients, care plans should be developed collaboratively with the patient, provider, and patient’s support system. Treatment decisions should be timely, based on evidence-based guidelines, and derived from individual preferences, risk factors, prognoses, comorbidities, and financial considerations.^8^ This initiative aimed to improve patient-centered diabetes screening and follow-up in a rural health care clinic to 80% within 8 weeks.

## Methods

SWCC is a rural primary care clinic in Grant County, Oregon. The clinic is staffed by 2 medical doctors and 2 nurse practitioners. The clinic sees an average of 50 patients per day and covers a population of 7200 people. The office support staff is composed of a clinical director, registered nurse, and 7 certified medical assistants. The payor sources are Medicare (35%), Medicaid (25%), private (34%), and dual Medicare/Medicaid (3%). The patient demographics are White (97%) and Hispanic (3%).

The Plan-Do-Study-Act was implemented, which is a systematic process for continued improvement and change. The goal of Plan-Do-Study-Act is to identify barriers, recognize areas that need change, and modify action to improve outcomes.^9^ This doctoral project met the U.S. Department of Health and Human Services definition of quality improvement and does not qualify as human subjects research; thus, an institutional review board was not required. No external funding was received for this initiative.

### Interventions

Two core interventions were implemented based on evidence-based practices that were used during the quality improvement initiative to improve patient-centered diabetes screening and follow-up: ADA diabetes screening and patient engagement (Table 1). For the screening intervention, the team screened patients using a standardized tool and initiated HbA1c orders through electronic medical records (EMRs) if indicated. A standard care log was used when reviewing lab results initiated by screening and patients who had HbA1c results >5.7% were indicated for a 3-month follow-up. Indicators were reviewed by providers and follow-up recommendations were provided based on patient history. Individuals newly diagnosed with prediabetes or diabetes, as well as those with suboptimal glycemic control based on changes in HbA1c from prior to most recent results, were scheduled for a 3-month follow-up. An adapted toolkit was used to implement the intervention of engagement for adult patients with diabetes (HbA1c > 6.5%; Table 1). The toolkit contained items based on guidelines that should be addressed on at least an annual basis, such as comprehensive eye, dental, and foot examinations, in addition to recommended lab testing and lifestyle modifications. Dot phrase templates were created and sent to all medical providers to incorporate into EMR documentation in order to streamline use.

**Table 1:** Core Interventions and Tests of Change

Core intervention	Plan-Do-Study-Act cycle			
	1	2	3	4
Prediabetes/diabetes screening	Implement prediabetes/diabetes screening	Modify ADA screening tool	Providers perform screening rather than CMAs	Provide screening handouts to patients
Patient engagement	Implement patient engagement screening	Create diabetes toolkit reminder	Simplify diabetes toolkit items for engagement	Add a toolkit template to EMR

Abbreviations: ADA, American Diabetes Association; CMA, certified medical assistant; EMR, electronic medical record.

### Measures

Two process measures and 2 outcome measures were evaluated (Table 2). The process measures were used to analyze the utilization of the screening criteria tool and patient engagement tool. Mean outcome scores were then calculated to determine the HbA1c testing and patient engagement percentages. The aim was determined by evaluating the response to the screening tool and initiating follow-up based on diagnostic results (Table 3). Patient satisfaction, based on after-visit surveys using a Likert scale (1 = *very dissatisfied*, 5 = *very satisfied*) was used as a balancing measure to ensure the team was effectively addressing patient needs.

**Table 2:** Core Intervention Measures and Results

Core interventions		Operational definitions	Baseline			PDSA 4			Project total		
Intervention	Tool		*N*	*n*	% or *M*	*N*	*n*	% or *M*	*N*	*n*	% or *M*
Prediabetes/diabetes screening	ADA screening criteria	Process: no. patients screened/no. patients who should have been screened		23		43	40	93	201	173	86
		Outcome: no. patients who had HbA1C/no. patients screened positive for risk factors and should have had a HgA1C	56	33	59	46	3	7	204	28	14
Patient engagement	Diabetes toolkit	Process: no. patients engaged/no. patients that should have been engaged	0	0	0	24	0	100	91	87	96
		Outcome: no. toolkit items that were completed/no. toolkit items = mean toolkit compliance %	0	0	0	0	0	97	0	0	94

Abbreviations: ADA, American Diabetes Association; HbA1C, hemoglobin A1c; PDSA, Plan-Do-Study-Act.

**Table 3:** Aggregate Summary Data from Standard of Care Log for Hemoglobin A1c Follow-Up After Screening

Indicator	Plan-Do-Study-Act cycle								
	1		2		3		4		Project total
	*n*	%	*n*	%	*n*	%	*n*	%	%
Did patients with prediabetes/diabetes have HbA1c ≥ 5.7%?									
Yes	15	75	25	78	39	85	27	90	83
No	5	25	7	22	7	15	3	10	18
Were patients with HbA1c ≥ 5.7% scheduled for follow-up appointments in 3 mo?									
Yes	14	93	21	84	37	95	24	89	91
No	1	7	4	16	2	5	3	11	9
NA	5	0	7	0	7	0	3	0	0

Abbreviations: HbA1c, hemoglobin A1C; NA, not applicable.

This table reflects the aggregate data for each indicator within the standard of care log. Each indicator was assigned an individual score based on the number of times that indicator was identified during chart audits for a particular Plan-Do-Study-Act cycle. These scores were then calculated cumulatively as a percentage for each indicator. Baseline per chart audit prior PDSA was 44%.

### Study of Interventions and Analysis

Prior to initiative implementation, a total of over 50 patient charts were audited using ADA criteria for diabetes screening. The most prevalent criterion applied was age ≥45 years. The chart audit included only patients who had been seen within the past 6 months. Laboratory values, along with relevant patient history, were reviewed to evaluate whether patients were appropriately screened for HbA1c testing in accordance with established guidelines. Additionally, follow-up care was assessed to determine if it met evidence-based recommendations. Analysis focused on identifying gaps in screening practices and adherence to follow-up protocols, ensuring alignment with current clinical standards.

Screening tools, satisfaction surveys, and engagement toolkits for people with diabetes were deployed and used to analyze qualitative data. Data collection occurred 3 times weekly and was used to analyze run charts as well as summary and aggregate tables. Run charts, which are used to monitor performance, trends or shifts over time, were analyzed for statistical significance by identifying signals of change with special-cause variation. Standard run chart rules were applied to analyze the data. Variation was evaluated by examining the number of runs to determine whether there was too much or too little variation. Shifts were defined as 6 or more consecutive data points falling on one side of the median. Trends were identified as 5 or more consecutive data points consistently increasing or decreasing. When no signals of special cause variation were detected, the data were interpreted as exhibiting common cause variation. Summary table data were analyzed to review mean outcomes, and qualitative data in the form of field notes were recorded 5 times weekly on aggregate data charts. Acquired data analysis and outcomes were discussed with clinical staff after each cycle to inform the test of change (TOC) for subsequent cycles. Preimplementation and postimplementation team surveys were conducted to assess team satisfaction.

## Results

The quality initiative aimed to improve patient-centered diabetes screening and follow-up in a rural primary care clinic to 80% in 8 weeks. Through implementation, the team improved the screening of 201 patients from baseline of 23% (*n* = 6 of 26) to 50% (*n* = 100 of 201; Fig. 1). The team also improved the follow-up of 128 patients with diabetes or prediabetes from a baseline of 44% (*n* = 12 of 27) to 83% (*n* = 106 of 128; Fig. 1). A balancing measure was implemented to assess patient satisfaction related initiative implementation. The team aimed to reach a patient satisfaction mean of 4.0, starting from a baseline score of 2.5. During the 8-week initiative, the team surveyed 86 of the patients that were engaged with toolkits. Patients engaged had the following demographics: White non-Hispanic (95%; *n* = 82 of 86) and Hispanic (5%; *n* = 4 of 86) Hispanic. Sixty-six percent (*n* = 57 of 86) of participants had Medicare, 17% (*n* = 15 of 86) had private insurance, 11% (*n* = 9 of 86) had Medicaid, and 6% (*n* = 5 of 86) had dual coverage Medicare/Medicaid, which is consistent with demographics of SWCC. At the end of implementation, the total initiative mean Likert score for patient satisfaction was 4.5.

**Fig. 1:**
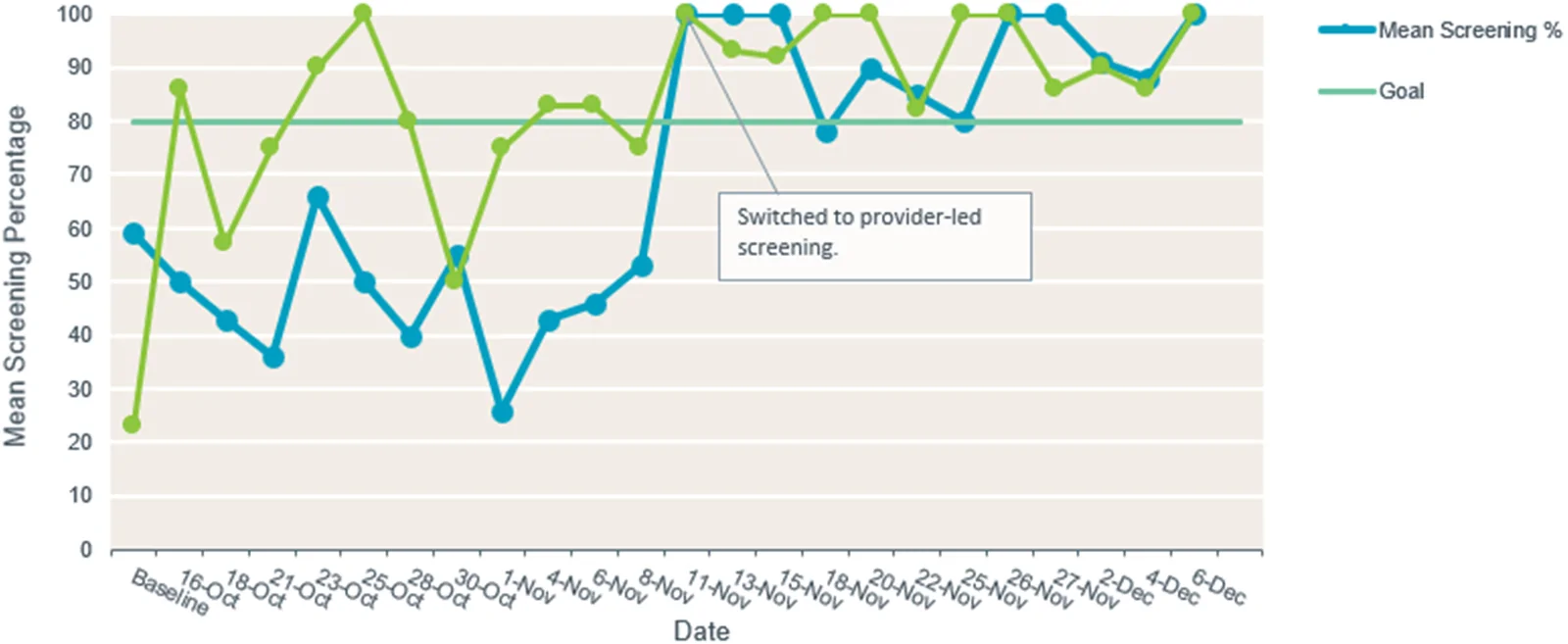
Analysis of utilization of American Diabetes Association screening criteria and diabetes screening scores on initial assessment. *Note*. Mean screening percentage = number of patients screened/number of patients who should have been screened (Blue). Mean HbA1c testing percentage = number of patients who had HbA1c testing/number of patients screened positive for risk factors and should have had a HbA1c test (Green).

### Prediabetes/Diabetes Screening

The screening tool was adapted based on updated ADA guidelines. During the 8-week period, 86% (*n* = 173 of 201) of the patients who met the criteria were screened, significantly improving the baseline of 23% (*n* = 13 of 56) by 50% (Table 2). Prior to project implementation, education was provided to clinical staff on guidelines and tools used during implementation were piloted in paper format. For cycles 1 and 2, screening was completed by certified medical assistants. In cycle 1, the mean screening percentage was 74% (*n* = 29 of 39). The TOC after cycle 1 was the modification of the screening tool for ease of use. Despite modifications, the mean screening percentage for cycle 2 was 70% (*n* = 26 of 37), lower than in the previous cycle. This led to the most impactful TOC, the initiation of provider-led screening. Provider-led screening in cycle 3 dramatically improved screening to a mean of 95% (*n* = 78 of 82). After cycle 3, statistical significance in the process measures was seen with 2 special-cause signals of change: a shift occurred on November 11th and an upward trend started on November 1st. For the TOC in cycle 4, self-screening tools were made accessible to patients in the clinical waiting areas to see whether the change led to patient-prompted screening. This TOC resulted in 0% of patients initiating conversation surrounding the need for screening. Despite the lack of results with patient-led prompting, the screening rate remained above goal in cycle 4 with a mean screening percentage of 93% (*n* = 43 of 46) for provider-led screening (Table 2). At the conclusion of cycle 4, there were 2 special cause signals in the outcome measure: an upward trend and a shift occurring on November 8th. Despite the team's progress, the process measure never met the goal of 80%, although it did improve by 50% as baseline data showed that 59% (*n* = 33 of 56) were not screened despite positive risk factors, whereas by the end of the initiative the total mean screening percentage revealed that only 14% (*n* = 28 of 234) of individuals who met screening criteria were not screened (Table 2). The outcome measure goal was achieved by improving from a baseline of 56% to 86% in cycle 4 with a total project mean of 80% (Table 2).

### Patient Engagement

The patient engagement toolkit was adapted from the ADA and was used to engage the population who had diabetes (HbA1c > 6.5%; Fig. 2). Cycle 1 revealed patient engagement of 82% (*n* = 18 of 22) with toolkit compliance of 93%. Despite success with initial engagement, providers struggled with implementation because of overburdened schedules. This led to the first TOC, which was to establish a provider reminder within the EMR for patients with diabetes. Patient engagement in cycle 2 was 95% (*n* = 21 of 22), with toolkit compliance of 86%, signifying a drop in toolkit engagement although it exceeded the initiative goal of 80%. Engagement items below the goal were reviewed to assess areas of deficiency. Items below goal were eye exams (67%; *n* = 14 of 21), foot exams (62%; *n* = 13 of 21), dental exams (67%; *n* = 14 of 21), weight loss advisement (67%; *n* = 6 of 9), physical activity (63%; *n* = 12 of 19), nutritional changes (71%; *n* = 10 of 14), and vaccination status (79%; *n* = 15 of 19). Results were discussed with providers and TOC for cycle 3 was to simplify the toolkit for use. Modification of the toolkit was the most impactful TOC during the 8-week initiative, with patient engagement for cycle 3 improving to 100% (*n* = 23 of 23), and toolkit item compliance rose to 98%. Analysis of run charts showed a stable process with appropriate variability. The final TOC for cycle 4 was to incorporate the toolkit compliance items into the EMR as a dot phrase template. In cycle 4, patient engagement was 100% (*n* = 24 of 24), and toolkit compliance was 97% (Table 2). In this cycle, the process measure showed 2 special-cause signals of change: an upward trend starting November 13th and a shift occurring on November 11th. There were also 2 special-cause signals of change in the outcome measure: an upward trend starting November 11th and a shift occurring on November 18th. This reflects statistical significance in both measures within the expected number for appropriate variability. The results of the initiative remained above goal throughout all 4 cycles, with a total mean patient engagement of 96% and total mean toolkit compliance of 94% (Table 2).

**Fig. 2:**
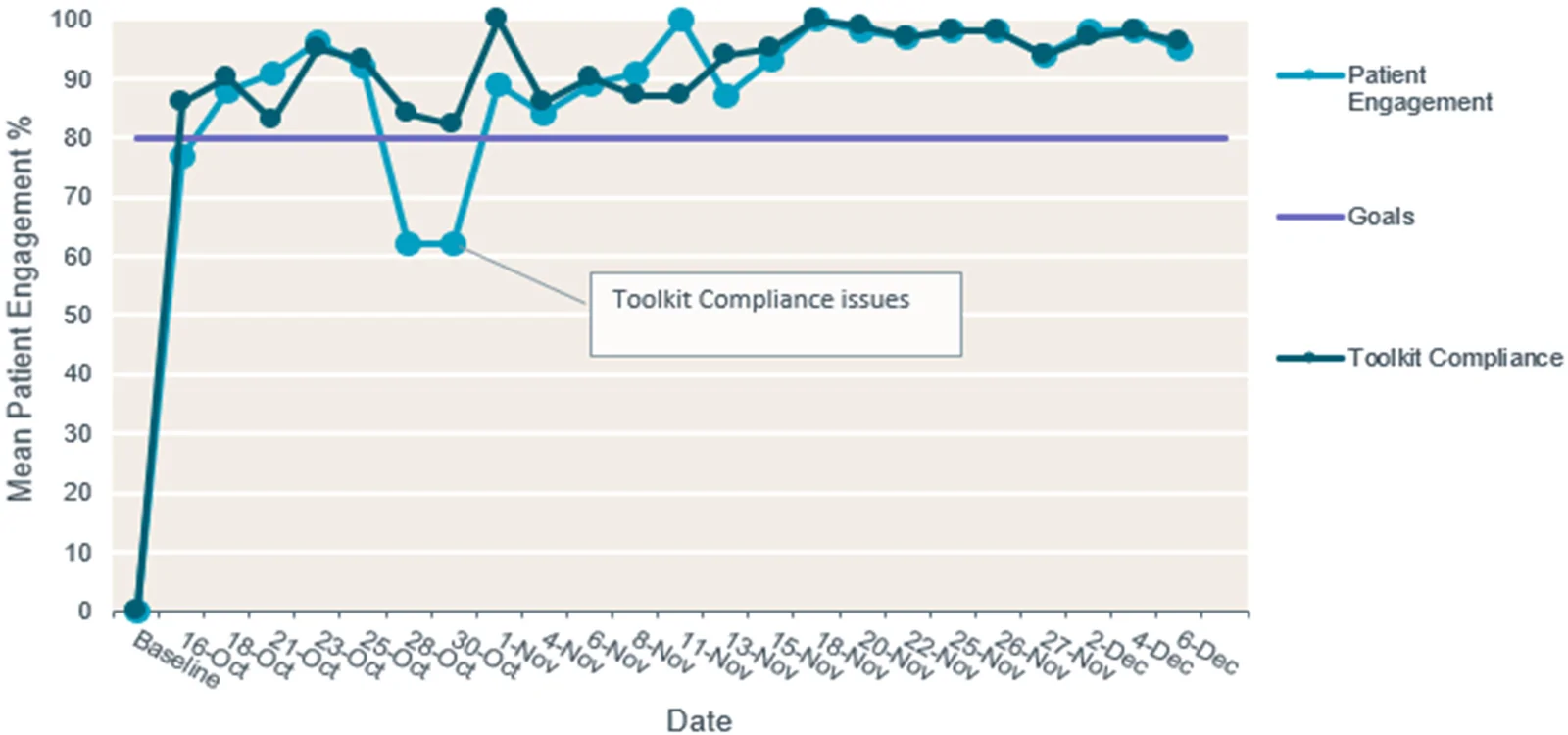
Analysis of patient engagement and toolkit item compliance. *Note*. Mean patient engagement percentage = number of patients engaged/number of patients that should have been engaged (Blue) mean toolkit compliance percentage = number of toolkit items that were completed/number of toolkit items that were required for the individual patient (Teal).

### Team Engagement

A Likert scale survey (1 = *very dissatisfied* to 5 = *very satisfied*) was used to assess employee perception of team engagement and organizational knowledge before and after initiative implementation. Preimplementation: when employees were asked about team cohesiveness, team members scored an average of 3.4. During implementation, the focus was to improve team morale, cohesiveness, and communication. Postimplementation survey results showed improvements, with team members scoring a mean of 4.8. Preimplementation employees were asked whether they understood their role in diabetes education, with team members scoring an average of 2.7. The focus here during implementation was to provide more education on diabetes and risk factors so that the team felt more comfortable addressing patient needs. Postimplementation survey results showed significant improvements, with team members scoring a mean of 5.0.

## Discussion

Analysis of data indicated that utilizing the diabetes screening tool improved follow-up and subsequently, treatment compliance rate for patients who had HbA1c results outside of the normal range. The rate increased from a baseline of 44% (*n* = 12 of 27) to a total initiative mean of 83%. Data also suggested that renewed education and use of updated screening guidelines improved patient screening by 50%. Eighty percent of the population who screened positively had HbA1c orders initiated. Patient engagement with a toolkit adapted for clinical use to all appearances resulted in overall mean engagement of 96%, with total initiative toolkit item engagement of 94%. Over the 8-week initiative, data analysis implies that patient satisfaction improved with patient-centered engagement from baseline Likert score of 2.5 to a total initiative mean Likert score of 4.7. Prioritizing follow-ups for individuals with HbA1c testing results of at least 5.7% seemingly improved individual health and minimized potential for risk factors. Providing educational opportunities for patients regarding their disease and risk factors while helping them become active participants in their health care likely bolstered provider-patient engagement. The strengths of this initiative are that the interventions were of little-to-no cost, and are adaptable for ease of incorporation in various health care settings.

### Interpretation

Implementing screening tool use within practice can impact individual health, community health, and associated costs. In accordance with Iragorri and Spackman^10^, evaluations with screening tools improve medical practice by providing evidence-based tools to inform care plan decisions. This improvement was evident in the increased mean follow-up percentage after screening initiation.

Communication practices between patients and providers are crucial to managing diseases and benefiting organizational health. According to Jenstad et al^11^, communication approaches directly impact patient outcomes, especially if approaches consider cultural barriers and are adapted to individuals based on potential impediments. Integration of toolkits into healthcare practice strengthens patient engagement in their health and fortifies education about their disease, promoting satisfaction surrounding care.

Implementing toolkits for people with diabetes improved patient-centered care by incorporating up-to-date, evidence-based guidelines. Thoele et al^12^ discovered that toolkits can improve plan implementation by using evidence-based clinical interventions to guide care plan implementation. They also stated that understanding clinical workflow can provide better direction for plan implementation and can allow development of an infrastructure to permit future management and sustain evidence-based interventions.

### Limitations

This qualitative initiative was limited by the practice size, given the area’s rurality and staffing shortage, and it was limited by the sample size, given the homogeneity of the general population. Implementation was conducted by 4 clinical providers, 3 of whom were only in the clinic on a part-time basis. Also, the project leader’s inexperience with quantitative analytical research inexperience might have been a limitation. Efforts made to minimize and adjust for the limitations included an attempt to have an adequate sample size despite constraints and homogeneity as well as obtaining expert guidance with data analysis throughout the initiative.

## Conclusions

During the 8-week initiative, patient-centered diabetes screening and follow-up improved from 44% to 86% using adapted evidence-based screening tools. Given the prevalence of diabetes in the country and the financial burden associated with it, medical practices must implement strategies to improve screening and follow-up to mitigate risk factors associated with diabetes, which may help to improve overall population health. The initiative’s success in patient care and team satisfaction demonstrates the potential for sustainability, and initiative tools can be easily adapted to other settings. Standardization of tools, continual educational opportunities to stay abreast of new and emerging guidelines and streamlining of EMR documentation processes are essential for further development and sustainability of this initiative. Further research should explore how early intervention and effective management of prediabetes and diabetes impact the development of comorbidities associated with the disease.

## Patient Consent

This project met the United States Department of Health and Human Services definition of quality improvement and does not qualify as human subjects research; therefore, an institutional review board was not required.

## Disclosure

The authors have no conflicts of interest to disclose.
